# Extraction of Bound Polyphenols from *Elaeagnus angustifolia* L. by Ultrasonic-Assisted Enzymatic Hydrolysis and Evaluation of Its Antioxidant Activity In Vitro

**DOI:** 10.3390/foods14091567

**Published:** 2025-04-29

**Authors:** Jingjing Lv, Lu Li, Zilong Liang, Wenyue Wu, Na Zhang, Qinghua Jia

**Affiliations:** 1 College of Food Science and Engineering, Tarim University, Alar 843300, China; 13333973910@163.com (J.L.); lilu9292jn@163.com (L.L.); lzlgtr2192@163.com (Z.L.); 13565116292@163.com (W.W.); 2 Production & Construction Group Key Laboratory of Special Agricultural Products Further Processing in Southern Xinjiang, Alar 843300, China; 3 Analysis and Testing Center, Tarim University, Alar 843300, China; qing_hua13@163.com

**Keywords:** *Elaeagnus angustifolia* L., bound polyphenols, UPLC–IMS-QTOF-MS, qualitative analysis, antioxidant activity

## Abstract

Herein, *Elaeagnus angustifolia* L. was utilized as a raw material to extract bound polyphenols using an ultrasound-assisted complex enzyme method for the first time. The effects of enzyme ratio, ultrasonic time, liquid-to-solid ratio, and pH value on the bound polyphenol yield were investigated using single-factor experiments. The key parameters were subsequently optimized using the Box–Behnken design. The optimal conditions identified were as follows: enzyme ratio (α-amylase/cellulase = 5:1 mg/mg), ultrasonic time of 50 min, liquid-to-solid ratio of 12:1 mL/g, and pH value of 5. Under these conditions, the bound polyphenol yield was measured at 13.970 ± 0.3 mg/g. A total of 27 phenolic compounds were identified using ultrahigh-performance liquid chromatography–ion mobility quadrupole time-of-flight mass spectrometry (UPLC–IMS-QTOF-MS), including two coumarins, five lignins, 10 polyphenols, nine flavonoids, and one tannin, and specifically containing Angeloylgomisin Q, Yakuchinone A, Furosin, 6-Dehydrogingerdione, and 4′-Methylpinosylvin, and so on. Antioxidant activity was assessed using the 1,1-diphenyl-2-picryl-hydrazil (DPPH) and 2,2′-azino-bis-(3-ethylbenzthiazoline-6-sulfonate) (ABTS) methods, revealing significant antioxidant potential. This study introduced a novel extraction process for bound polyphenols from *E. angustifolia* L. and provided the first qualitative analysis of bound polyphenols in this species, establishing a scientific foundation for its development and application in the functional food, medicine, and cosmetics industries.

## 1. Introduction

*Elaeagnus angustifolia L.*, commonly referred to as Qilixiang, Guixiangliu, and Jigedai, belongs to the Elaeagnaceae family. Currently, over 80 recognized varieties of *E. angustifolia* L. exist worldwide, primarily distributed across northwest China, subtropical Europe, parts of North America, and the Himalayas [1,2,3,4]. One of the prevalent varieties of *E. angustifolia* L. can be found in Xinjiang. This species is characterized by its drought tolerance, saline–alkali resistance, and ability to withstand wind and sand. Additionally, nitrogen-fixing rhizobia associated with the roots of *E. angustifolia* L. contribute to soil fertility and enhance the soil environment [5,6]. Thus, *E. angustifolia* L. is recognized as a significant tree species for semiarid regions and saltland shelterbelts. The pulp of *E. angustifolia* L. is rich in various nutrients, including starches [7], sugars, fats, proteins, amino acids, and minerals [8,9]. Recent studies indicate that the fruit of *E. angustifolia* L. is rich in sugars, tannins, flavonoids, minerals, and various trace elements, exhibiting health benefits such as hypoglycemic, cholesterol-lowering, anti-inflammatory, and antioxidant effects [10].

Polyphenols are secondary metabolites widely distributed in plants, containing an aromatic ring and one or more hydroxyl groups. Based on the number and position of the hydroxyl groups, they can be classified into phenolic acids, flavonoids, stilbenes, and lignin [11]. In recent years, many reports have documented the biological activities of plant polyphenols, such as antiviral, antibacterial, antitumor, hypoglycemic, and hypolipidemic [12,13]. Phenolic compounds can be divided into free polyphenols (FPs) and bound polyphenols (BPs) based on their dissolution characteristics [14,15]. FP is mainly present in the food matrix as monomers and is easily dissolved in water and organic solvents [16]. BP is often present in the plant cell wall matrix and is linked to macromolecules such as cellulose, structural proteins, and cell wall polysaccharides in the form of monomers, dimers, or oligomers through ester bonds, ether bonds, or C–C bonds. Such polyphenols are more difficult to extract [17]. BP mainly exists in the residues of FP released by water and is not released through the hydrolysis process. Thus, most people often ignore BP. Existing studies have shown that the BP content in plants is significantly higher than the FP content, and BP has superior biological activities [18,19].

The methods for extracting BP from plants include chemical methods, biological methods, and physically assisted extraction methods. Chemical methods usually require a longer extraction time and a more complex pretreatment process and need to be performed under high-temperature conditions, which will lead to the degradation of some phenolic compounds [20]. The enzymatic hydrolysis reaction has the advantages of being green, mild, and efficient [21]. In the food industry, physical assistive technology has been widely used, with the advantages of high mass transfer efficiency and environmental friendliness [22,23]. It has been reported that the combination of ultrasound with other hydrolysis methods such as alkaline hydrolysis and supercritical fluid extraction is more effective in releasing BP than any other single extraction method [24,25], showed that the amount of tartary buckwheat BP released by ultrasound-assisted hydrolysis was significantly higher than that released by acid–base hydrolysis. Compared with previous studies, in this study, the combination of ultrasonic extraction and enzymatic hydrolysis was used to extract BP from *E. angustifolia* L. The ultrasonic extraction method utilizes the cavitation effect, mechanical effect, and thermal effect generated by ultrasonic waves to disrupt the plant cell wall, thereby accelerating the infiltration of solvents and the dissolution of active ingredients. The enzymatic hydrolysis method has the advantages of environmental protection and mildness. Compared with traditional extraction methods, the combination of the two can achieve efficient extraction and maintain mild reaction conditions and environmental friendliness.

In this experiment, ultrasound-assisted enzymatic hydrolysis was used for the first time to extract BP from *E. angustifolia* L. from Xinjiang, China. The effects of the compound enzyme ratio, ultrasonic time, liquid-to-solid ratio, and pH on BP yield were investigated. The process parameters were optimized using the response surface methodology. In addition, we also analyzed the antioxidant activity in vitro of BP from *E. angustifolia* L., and identified the phenolic compounds by UPLC–IMS-QTOF-MS, aiming to provide new ideas for its application in the research and development of functional foods, drugs, and cosmetics.

## 2. Results and Discussion

### 2.1. Single-Factor Investigation

#### 2.1.1. Effect of Enzyme Ratio on BP Extraction

**Figure 1:**
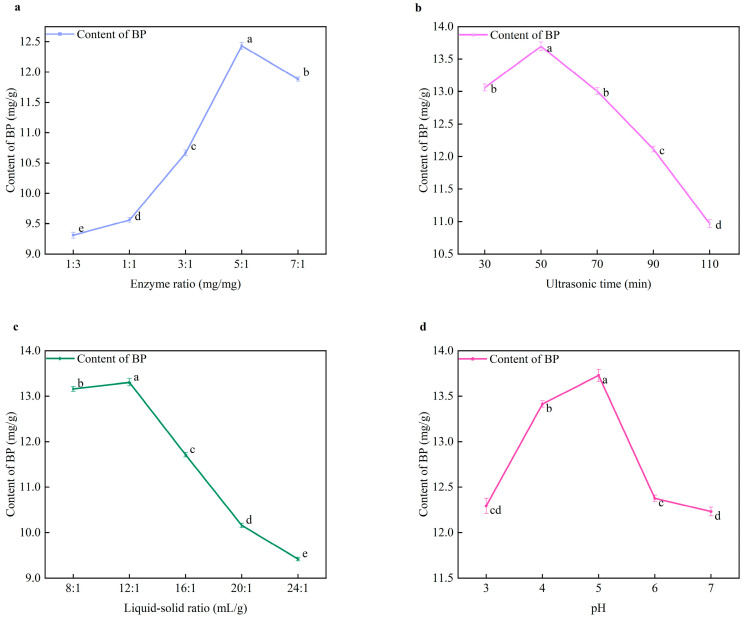
Impact of various operating parameters on BP content: (**a**) effects of enzyme ratio; (**b**) ultrasound time; (**c**) liquid/solid ratio; (**d**) pH on the yield of BP. (Different letters represent significant differences, *p* < 0.05).

The effect of the enzyme ratio on the extraction rate of *E. angustifolia* L. BP is shown in Figure 1a. After the preliminary test, α-amylase and cellulase were selected. The addition ratio of the two enzymes was increased from 1:3 to 5:1 mg/mg, and the BP yield showed an upward trend, reaching a maximum when the enzyme ratio was 5:1 mg/mg. When the enzyme ratio continued to increase to 7:1 mg/mg, BP showed a downward trend. BP is often present in the plant cell wall matrix and is linked to starch and other macromolecules in the form of monomers, dimers, or oligomers through ester bonds, ether bonds, or C–C bonds [26]. Amylase can break the starch chain, destroy its spatial structure, and hydrolyze the glycosidic bond so that the phenolic substances embedded in the starch network structure can be released. Cellulase can destroy the cell wall structure because the cell wall structure is dominated by cellulose and hemicellulose, and the phenolic substances connected to it are continuously released into the solvent, thereby promoting the extraction efficiency of phenolic substances [27]. In this experiment, with an increase in the amount of α-amylase, the BP yield in *E. angustifolia* L. increased continuously, which may be due to the combination of BP and starch in *E. angustifolia* L. When it increased to a certain amount, it showed a downward trend, which might be because the enzyme concentration and substrate reached saturation, but the extraction rate decreased. Some researchers have shown [28] that enzyme concentration and substrate saturation have an effect. Simply put, when α-amylase is excessive, it may preferentially occupy the reaction interface, resulting in the inability of cellulase to effectively contact cell wall substrates, or the oligosaccharides generated by α-amylase to decompose starch may inhibit itself or cellulase activity and reduce overall efficiency. Therefore, considering the above reasons and cost issues, the best enzyme ratio is 5:1.

#### 2.1.2. The Influence of Ultrasound Time on BP Extraction

The effect of ultrasonic time on the BP extraction rate of *E. angustifolia* L. is shown in Figure 1b. The BP yield increased with an increase in ultrasonic time from 30 to 50 min and reached the peak at 50 min, but it decreased sharply with an increase in extraction time. This may be because these compounds need a certain amount of time to dissolve and diffuse from the plant cell membrane to the solvent medium under the mechanical action of the ultrasound, and the chemical bonds connected to BP cannot be completely broken in a short time. Prolonging the extraction time can increase the extraction amount of BP. However, with the extension of time, some of the dissolved polyphenols are prone to oxidation and loss, and the structures of some unstable components are easily destroyed under long-term ultrasound, and a large number of impurities are dissolved, which is not conducive to separation and extraction [29,30,31]. It is also possible that a very long ultrasonic time will reduce the activity of the enzyme, resulting in a decrease in BP yield [32]. In this experiment, the highest BP yield was obtained at 50 min, which was consistent with the experimental results reported by Liu and Jiao et al. [33,34]. Therefore, the appropriate ultrasonic time is 50 min.

#### 2.1.3. The Effect of Liquid-to-Solid Ratio on BP Extraction

The effect of the liquid-to-solid ratio on the BP extraction yield of *E. angustifolia* L. is shown in Figure 1c. The BP yield increased slowly with an increase in the liquid-to-solid ratio from 8:1 to 12:1 mL/mg, and it reached its maximum when the liquid-to-solid ratio was 12:1 mL/mg. The reason may be that the liquid-to-solid ratio was small and the reaction between the powder of *E. angustifolia* L. and the solvent was not sufficient, which affected the dissolution of BP, indicating that the increase in liquid-to-solid ratio promoted the hydrolysis and release of BP by the enzyme. However, if we continue to increase the solid-to-liquid ratio, the BP yield will show a serious downward trend. This may be because of the increase in the volume of the liquid, which increases the contact area with the air, ensuring that the released BP is oxidized, or it may be because the amount of enzyme and the reaction substrate are saturated. If we continue to increase the liquid-to-solid ratio, it will not only increase the solvent consumption but also may dissolve more alcohol-soluble impurities such as pigments [35,36]. This will increase the workload and cost of subsequent processes; the test results are consistent with the trend reported by Gao et al. [37]. Therefore, the optimum liquid-to-solid ratio is 12:1 mL/mg.

#### 2.1.4. Effect of pH on BP Extraction

The effect of pH on the BP extraction rate of *E. angustifolia* L. is shown in Fig. 1d. When the pH was less than 5.0, the BP yield increased significantly with an increase in pH value. When the pH of the enzymatic hydrolysate was 5.0, the BP yield reached the maximum. When the pH was greater than 5.0, the BP yield decreased with an increase in pH, indicating that the enzyme activity was affected by the pH value. The complex enzyme activity was strong under the weak acid condition of pH 4–5, which is conducive to the release of BP, and the complex enzyme activity was inhibited under the strong acid and neutral conditions, which was not conducive to its role [38,39]. The pH value not only affects the enzyme activity but also affects the activity of polyphenols. Previous studies [40] have shown that most polyphenols are structurally stable in the pH range of 2–5, and hydrolysis or decarboxylation occurs under alkaline conditions, resulting in structural damage. Therefore, the optimal enzyme solution pH is 5.0.

### 2.2. Response Surface Methodology for Optimizing the BP Extraction Process of E. angustifolia L.

Based on single-factor test results, the four factors of enzyme ratio (A), time (B), liquid-to-material ratio (C), and pH (D) were selected as variables, and the BP yield of *E. angustifolia* L. was used as the response value (Y). According to the principle of the Box–Behnken experiment, we designed a response surface experiment to optimize the technological parameters of *E. angustifolia* L. BP. Table 1 illustrates the design and results of the experiment. The experimental results were analyzed and fitted, and the quadratic regression equation model of the relationship between polyphenol yield (*Y*) and each factor variable was established:

$$\begin{array}{l} Y=14.01-0.2854A+0.2815B-0.4264C+0.0073D+0.0897AB-0.3029AC-\\ 0.5984AD+0.3084BC+0.2141BD-0.2471CD-1.10A^{2}-0.7603B^{2}-0.9072C^{2}\\ -0.9319D^{2}. \end{array}$$

The regression model was comprehensively evaluated using analysis of variance, and results are shown in Table 2. The regression model of F = 12.68, *p* < 0.0001, misfit term *p* = 0.2654 > 0.05 loss of fit was not significant, and the coefficient of determination R2 = 0.9269. Therefore, the fitting degree of the regression model is very good, which can be used to analyze and predict the process of the ultrasound-assisted enzymatic extraction of *E. angustifolia L.* BP. In the regression model, the regression coefficients of C, A, and B were significant (*p* < 0.05). The C item was extremely significant (*p* < 0.01), and the quadratic item (A^2^, B^2^, C^2^, D^2^) showed a significant level (*p* < 0.01). AD was the same as the quadratic item (*p* < 0.01), and the other items had no significant effect on the extraction rate of BP (*p* > 0.05). The mechanism of the four variables on BP yield is as follows: liquid-to-solid ratio (C) > enzyme ratio (A) > time (B) > pH (D).

**Figure 2:**
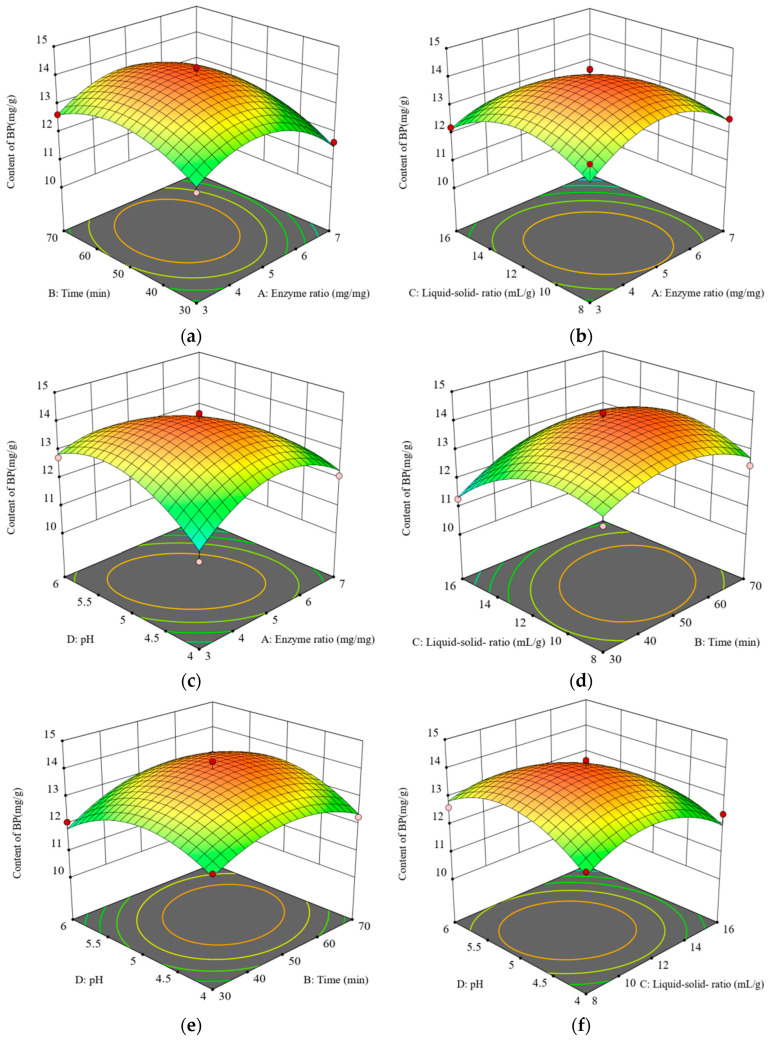
Effects of interaction of factors on BP yield. (**a**) Enzyme ratio/time. (**b**) Enzyme ratio/liquid/solid ratio. (**c**) Enzyme ratio/pH. (**d**) Time/liquid/solid ratio. (**e**) Time/pH. (**f**) Liquid/solid ratio/pH.

The response surface and contour lines of each interaction are shown in Figure 2. The response surface and contour map intuitively reflect the influence of the interaction of two factors on the BP extraction rate of *E. angustifolia* L. With the increase in the slope of the response surface, the contour line becomes denser, indicating that each factor has a great influence on the leaching rate of *E. angustifolia* L. BP. The contour map is an ellipse, indicating that there is an interaction between the two factors. The closer to the circle, the smaller the interaction. The color in the response surface fades from green to red, indicating an increase in the response value. The red area corresponds to the highest extraction rate, and the green area corresponds to the lowest value. The darkest or brightest areas, such as the red highlights, often indicate the global optimal combination of parameters.

According to the quadratic regression model established by response surface analysis, the optimum extraction conditions of *E. angustifolia* L. BP were as follows: enzyme ratio, 4.76:1; ultrasonic time, 52.97 min; liquid-to-solid ratio, 11.93:1 mL/g; and pH, 5.087. The BP yield was 14.093 mg/g. According to the actual operation, the parameters were adjusted as follows: enzyme ratio of 5:1, ultrasonic time of 50 min, liquid-to-solid ratio of 12:1 mL/g, and pH of 5. Five parallel experiments were conducted according to these process conditions. The BP yield was 13.970 ± 0.3 mg/g, which was consistent with the predicted value given by the model. Therefore, the model was feasible, and the optimized process parameters were accurate and reliable.

### 2.3. Determination of Phenols

#### Phenol Content of *E. angustifolia* L.

The standard regression equation and correlation coefficient of the combined polyphenols of *E. angustifolia* L. are shown in Table 3, and results show that the linear relationship with the components is good.

**Figure 3:**
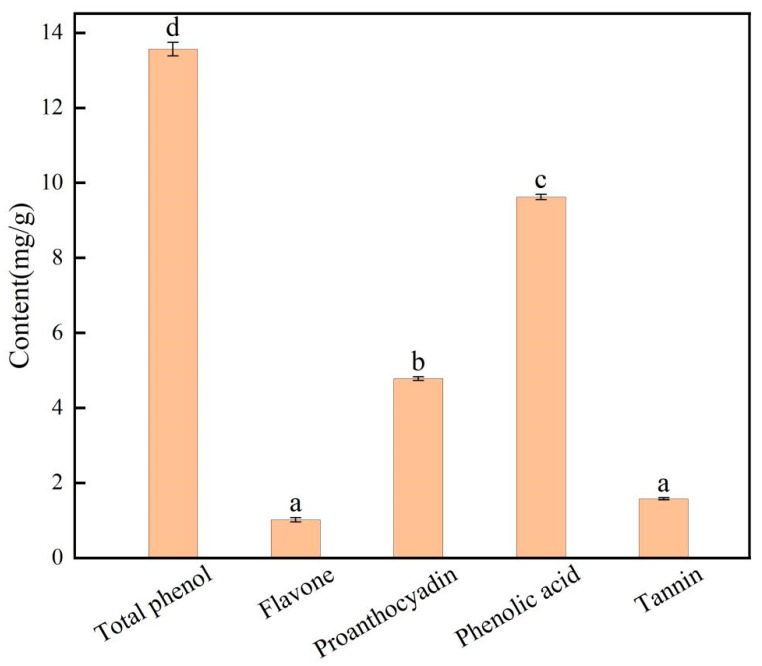
The content of bound phenols in *E. angustifolia* L. (Different letters represent significant differences, *p* < 0.05).

The phenolic compound content of *E. angustifolia* L. is shown in Figure 3. After the response surface optimization test, the polyphenol content of *E. angustifolia* L. BP was up to 13.566 mg/g. The phenolic acid content was 9.622 mg/g, and the proanthocyanidin, tannin, and flavonoid contents were 4.783, 1.575, and 1.015 mg/g, respectively. Results showed that *E. angustifolia* L. is rich in polyphenols, imparting it with good biological function and antioxidant activity.

### 2.4. Qualitative Analysis of BPs of E. angustifolia L.

**Figure 4:**
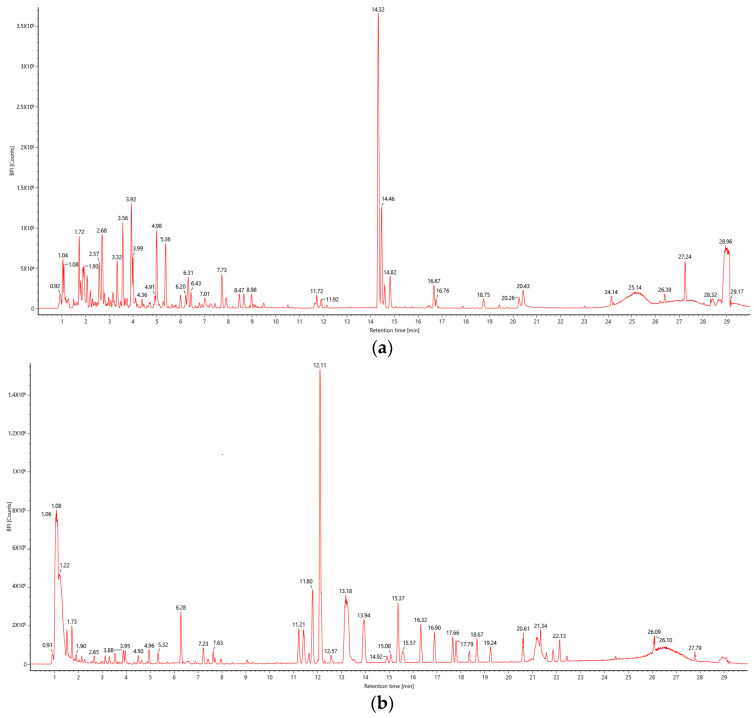
UPLC–IMS-QTOF-MS total ion diagram of *E. angustifolia* L. extract. (**a**): Positive ion mode; (**b**): negative ion mode.

The chemical constituents of the extract were studied by UPLC–IMS-QTOF-MS, and the first-order total ion flow map in ion mode was obtained, as shown in Figure 4. The results obtained by the parent ion mass-to-charge ratio, the ion information of the characteristic fragments, and the reference to the relevant literature [41,42] are shown in Table 4. A total of 27 compounds were identified in the BP freeze-dried powder of *E. angustifolia* L., including two coumarins, five lignins, 10 polyphenols, nine flavonoids, and one tannin.

It is well known that Angeloylgomisin Q is a kind of lignin, as shown in Table 4. Some researchers [43,44] have concluded that Angeloylgomisin Q shows moderate activity in anti-HIV-1 activity screening, and may play an antiviral role by interfering with the viral replication cycle. Lin Rong-Jyh et al. showed [45] that Yakuchinone A was the main component of A.oxyphylla, which had anti-inflammatory, antitumor, antibacterial, and gastric protective activities. Ji Yun Baek et al. [46] found that Furosin could scavenge free radicals and inhibit apoptosis, protecting HT22 neurons from glutamate-induced oxidative damage. Some researchers [47] had found that 6-Dehydrogingerdione can activate the Keap1-Nrf2-ARE pathway, up-regulate glutathione synthase and HO-1, protect PC12 cells from oxidative damage, and has good antioxidant activity. It can be seen that BP extracted from *E. angustifolia* L. was an active ingredient with multiple active functions. This is conducive to opening up the market in the food, pharmaceutical, and cosmetics industries.

### 2.5. Antioxidant Analysis of E. angustifolia L. BP Extracts In Vitro

**Figure 5:**
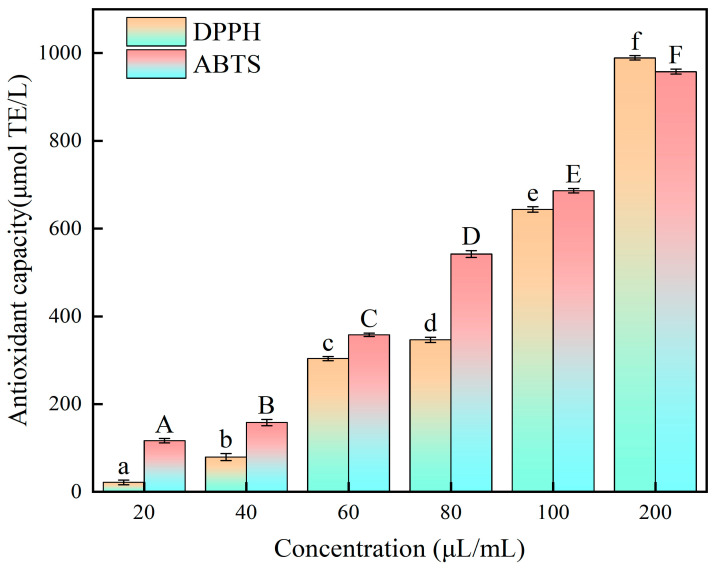
Scavenging ability of DPPH and ABTS by BP extract of *E. angustifolia* L. (Different letters represent significant differences, *p* < 0.05).

The scavenging ability of the BP extract on DPPH and ABTS free radicals is shown in Figure 5. As the concentration of the BP extract increased, the scavenging ability increased. When the concentration of the BP extract was 20–100 μL/mL, the scavenging ability of ABTS was higher than that of DPPH. This may be because the ABTS free radical has higher water solubility and electron transfer ability and the DPPH free radical is more stable [48]. The ABTS radical is more easily formed in aqueous solution. Therefore, its scavenging ability may be more affected by the water solubility of polyphenols [49,50]. When the addition amount was 200 μL/mL, the scavenging ability of DPPH was higher than that of ABTS. The different values obtained by different free radicals depend on the polarity and symbiotic mechanism of the compounds. More importantly, the mechanisms of different free radicals are different [51], which are the potential reasons for this situation. For the ABTS radical, phenolic compounds reduce free radicals through single electron transfer (SET) or hydrogen atom transfer (HAT) mechanisms [52]. Phenolic compounds act as electron donors and directly transfer electrons to ABTS to form stable phenolic oxygen radicals and reduced ABTS [53]. For the DPPH free radical, phenols can transfer electrons first, then release protons, and can also be transferred through HAT. The phenolic hydroxyl group directly provides hydrogen atoms to terminate the free radical chain reaction. Most importantly, phenols first dissociate protons in the solvent to form anions, and then transfer electrons [51]. All of the above are the possible mechanisms of phenols in the DPPH free radical reaction.

It is a common method to evaluate antioxidant capacity with ABTS and DPPH. Lu et al. [54] determined the DPPH free radical scavenging activity of onion BP, and the results were 447.14–623.95 μmol TE/g. Qin et al. [55] determined that the IC_50_ values of DPPH and ABTS+ were 0.0599 and 0.0121 mg/mL, respectively. Xiao et al. [56] determined the DPPH and ABTS scavenging ability of different varieties of rice bran BP, and the DPPH and ABTS scavenging ability ranges were 3.97–6.68 and 7.8–11.8 mgVC/gDW, respectively. In general, with an increase in polyphenol concentration, the scavenging rates of DPPH and ABTS free radicals gradually increase. In a lower concentration range, the clearance rate increases significantly with an increase in concentration. In a higher concentration range, the clearance increases slowly with an increase in concentration [57]. When the additional amount of BP extract was 200 μL/mL, the scavenging ability of DPPH and ABTS was as high as 989.28 and 957.86 μmol Trolox/L, respectively. This indicates that BP has good antioxidant activity.

## 3. Materials and Methods

### 3.1. Materials and Reagents

Xinjiang *E. angustifolia* L. samples were collected in 2024. α-Amylase (3700 U/g), cellulase (50 U/mg), Folin phenol solution, vanillin, and anhydrous sodium carbonate were obtained from Tuckblue Biotechnology Co., Ltd. (Alar, China). In addition, gallic acid, proanthocyanidins, Trolox standards, and rutin were provided by Sigma. Anhydrous ethanol, sodium nitrite, aluminum nitrate, NaOH, DPPH, ABTS, etc., were provided by Dingyuan Biotechnology Co., Ltd. (Alar, China).

### 3.2. Test Instruments

UPLC–IMS-QTOF-MS (Waters, Milford, MA, USA), a UV–visible spectrophotometer (J6, Shanghai Jinghua, Shanghai, China), an rE-3000A rotary evaporator (Xi’an Hepu, Xianxi, China), a TGL-20B high-speed desktop centrifuge (Shanghai anting, Shanghai, China), an SB-5200DT ultrasonic cleaning machine (Guangzhou Huruiming Instrument Co., Ltd., Hangzhou, China), a pilot3–6 m automatic silicone oil circulation system freeze-drying machine (Beijing Boyikang Experimental Instrument Co., Ltd., Beijing, China), a Synergy H1 multifunction microplate reader (BIO-TEK, Palo Alto, CA, USA), and an LE2002E electronic balance were used in the experiments (Satorius, Gottingen, Germany).

### 3.3. Removal of FP from E. angustifolia L.

To avoid the influence of FP in *E. angustifolia* L. on the analysis of the results, it should be extracted completely before extracting BP. According to the method described in literature reference [58], *E. angustifolia* L. was dried in a 45 °C drying oven, and the core was removed. *E. angustifolia* L. pulp was ground into powder and sieved by a 40 mesh screen, and refrigerated. Dry powder (200 g) was added to 75% ethanol at a liquid-to-solid ratio of 20:1 mL/g. After ultrasonication at room temperature (25 °C) for 30 min, the extract was centrifuged at 8000 r/min for 10 min, and the precipitate was repeatedly extracted more than three times according to the above steps until the extract did not contain phenolic substances, using the Folin–Ciocalteu method. The supernatant was collected, and the precipitate was recovered and dried at 40 °C.

### 3.4. Extraction of BP from E. angustifolia L.

With reference to the method of previous studies [59], slightly modified, BP was extracted. *E. angustifolia* L. without FP (1 g) was accurately weighed and hydrolyzed with a certain amount of compound enzyme solution. After ultrasonic extraction at a certain temperature for a period of time, it was centrifuged (8000 r/min, 10 min), the precipitate was extracted twice again, and the supernatant was combined for later use.

### 3.5. Design of Single-Factor Experiment for BP Extraction of E. angustifolia L.

According to the previous test, the ultrasonic temperature should be controlled at 50 °C, and the control variable method was used. The fixed factor level involved an enzyme ratio of α-amylase/cellulase = 5:1 mg/mg, an ultrasonic time of 50 min, a liquid-to-solid ratio of 12:1 mL/g, and pH 5. The effects of different enzyme ratios (1:3, 1:1, 3:1, 5:1, and 7:1 mg/mg), ultrasonic times (30, 50, 70, 90, and 110 min), liquid-to-solid ratios (8:1, 12:1, 16:1, 20:1, and 24:1 mL/g), and pH (3, 4, 5, 6, and 7) on the BP yield from *E. angustifolia* L. by ultrasound-assisted enzymatic extraction were investigated.

### 3.6. Experimental Design of Response Surface

Based on a single-factor test, the optimal factors and levels were selected. The enzyme ratio (A), time (B), liquid-to-solid ratio (C), and pH (D) were used as independent variables, and the BP yield of *E. angustifolia* L. (Y) was used as the target value. A response surface optimization test of four factors and three levels was designed. The levels of the experimental factors are shown in Table 5.

### 3.7. Determination of Phenolic Compound Content

#### 3.7.1. Determination of Total Phenol

The total phenol content was determined by the Folin–Ciocalteu colorimetric method [60]. Here, 0.1 mg/mL gallic acid standard solution (0, 0.1, 0.2, 0.3, 0.4, 0.5, and 0.6 mL) was accurately measured in a 25 mL colorimetric tube, added with distilled water to 10 mL, added with 0.3 mL of Folin–Ciocalteu reagent, shaken well, and allowed to stand at room temperature for 5 min. Then, 1 mL of 15% sodium carbonate solution was added and shaken well. Absorbance was measured at 746 nm after reaction at 25 °C for 2 h in the dark. A standard curve was plotted with the mass concentration of gallic acid (X) as the abscissa and the absorbance value (Y) as the ordinate.

The extract was diluted to obtain the sample solution to be tested. The absorbance of 0.2 mL of the solution was measured using the abovementioned method, and the BP content was calculated using the following formula:

$$Y=\frac{c\times D\times V\times 10^{-3}}{m}.$$

where *Y* represents BP content (mg/g), *c* is the mass concentration of the test solution (μg/mL), *D* stands for dilution multiple, *V* is the sample liquid volume (mL), and *m* is the mass of the raw material quality (g).

#### 3.7.2. Flavone Determination

Determination of flavonoids was determined using the method described by Zhao et al. [61]. The 0.15 g/L rutin standard solution was added with 0, 1.0, 2.0, 3.0, 4.0, 5.0, and 6.0 mL, respectively. Distilled water was added to the test tube to 6 mL, and 1 mL of 5% sodium nitrite was added to each test tube for 6 min. Then, 1 mL of 10% aluminum nitrate solution was added for 6 min of reaction. Finally, 4% sodium hydroxide was added to make the volume of 25 mL, and the absorbance was measured at 510 nm. Taking the rutin (X) mass concentration as the horizontal axis and absorbance (Y) as vertical axis, the standard curve was plotted to get a straight line. Then, the standard curve was used for regression, and the content of total flavonoids in the sample was obtained.

#### 3.7.3. Determination of Proanthocyanidins

Total proanthocyanidins were determined using the hydrochloric acid–vanillin method, as described by Lv et al. [62], with slight modifications. Then, 1 mg/mL proanthocyanidin standard solution was diluted into five gradients of 0.5, 0.4, 0.3, 0.2, and 0.1 mg/mL, and 1 mL of each gradient standard solution was accurately measured and added to a mixture containing 6 mL of vanillin–methanol solution and 3 mL of concentrated hydrochloric acid. The mixture was quickly mixed and reacted for 20 min under dark conditions. Absorbance was measured at a wavelength of 500 nm. The blank control was anhydrous methanol. A standard curve was drawn with the mass concentration of proanthocyanidin (X) as the abscissa and the absorbance value (Y) as the ordinate. The proanthocyanidin content in the sample extract was calculated using the standard curve regression equation.

#### 3.7.4. Determination of Phenolic Acid

The Kumaran et al. [63] method was slightly modified, and the gallic acid standard at 1.0 mg/mL was diluted with five gradients (0.2, 0.4, 0.6, 0.8, 1.0 mg/mL). A series of concentration gradients of gallic acid reference solution 0.1 mL, Folin phenol reagent 0.5 mL, and 20% sodium carbonate 1.5 mL were mixed evenly, and then diluted with distilled water to a 10 mL volumetric flask, placed at 25 °C for 2.0 h, using distilled water as a blank, and the absorbance was measured at a wavelength of 765 nm. The content of phenolic acid was obtained by fitting the standard curve with the gallic acid content (X) as the horizontal axis and the absorption rate (Y) as the vertical axis.

#### 3.7.5. Determination of Total Tannins

The tannin content was determined using the hydrochloric acid–vanillin method, with reference to the slightly modified method reported by Hao et al. [64]. Then, 0, 1.0, 2.0, 3.0, 4.0, and 5.0 mL of 1 mg/mL catechin standard solution were accurately removed and diluted to 10 mL with methanol solution. Each 0.5 mL was transferred to six test tubes wrapped with aluminum platinum paper, 3 mL of 4% vanillin–methanol solution, and 1.5 mL of concentrated hydrochloric acid were added and reacted at 20 °C for 20 min, and absorbance was measured at a wavelength of 510 nm. A standard curve was drawn with catechin mass concentration (X) as the abscissa and absorbance value (Y) as the ordinate, and the tannin content in the sample extract was calculated using the regression equation of the standard curve.

### 3.8. Qualitative Analysis of BP in E. angustifolia L.

Referring to previous research [65], the qualitative analysis of the BP component was slightly modified. Here, 10 mg of freeze-dried *E. angustifolia* L. BP powder was accurately weighed, and 1 mL of purified water and 1 mL of mass spectrometry methanol were added. The mixture was shaken to completely dissolve the powder and centrifuged at 10,000 rpm for 10 min. UPLC–IMS-QTOF-MS analysis was performed after filtration using a 0.22 μm filter membrane.

The chromatographic conditions were as follows: An ACQUITY UPLC-BEH C18 chromatographic column (2.1 mm × 100 mm; particle size, 1.7 μm) was used. Mobile phase A was acetonitrile/methanol = 9:1, and mobile phase B was 0.1% formic acid aqueous solution. The column temperature was 25 °C. The volume of injection was 1 μL. The mobile phase elution conditions were 0–27 min, 95% B; 27–28 min, 100% B; 28–28.1 min, 100% B; and 28.1–30 min, 95% B.

The mass spectrometry conditions were as follows: An ESI ionization source and positive and negative ion modes were used. The capillary voltage was 2.5 kV, and the cone hole voltage was 40.0 V. The desolvation temperature was 500 °C; the relative molecular mass scanning range (*m*/*z*) was 50–2000; the scanning time was 0.2 s; the cone gas flow rate was 50 L/h; the desolvation gas flow rate was 100 L/h; the ion source temperature was 120 °C; and the desolvation gas temperature was 500 °C.

### 3.9. Study on Antioxidant Activity In Vitro of BP Extract of E. angustifolia L.

#### 3.9.1. DPPH Radical Scavenging Assay

According to the reports by Li and Sathasivam [66,67], extracts of different concentrations were prepared, and 100 μL of each extract was added to 3.9 mL of 25 mg/L DPPH solution. Afterward, absorbance was measured at 517 nm for 20 min in the dark. Trolox was prepared with 0–1000 μmol/L Trolox, and the DPPH free radical scavenging activity was calculated by the standard curve. The regression equation of the standard curve was obtained: Y = −0.0006X + 0.6459, R^2^ = 0.9946. The results were expressed as μmol Trolox/L.

#### 3.9.2. Determination of ABTS Free Radical Scavenging Ability

The ABTS radical scavenging ability test was slightly modified by referring to the method reported by Dong et al. [68]. The ABTS cation radical was prepared by reacting ABTS stock solution (7 mmol/L) with potassium persulfate (2.45 mmol/L) at room temperature for 12–16 h in the dark. The ABTS free radical solution was diluted with methanol to reach an absorbance of 0.70 ± 0.02 at 734 nm. Samples (100 μL) with different solubilities were prepared and placed in 3.9 mL of ABTS solution, and absorbance was measured at 734 nm after reaction for 8 min in the dark. The standard curve of ABTS free radical capture ability was prepared and analyzed by preparing a 0–1000 μmol/L Trolox standard sample. The results show that the fitting results of the model are Y = −0.0007X + 0.7015, R^2^ = 0.9981; the results were expressed with μmol Trolox/L.

### 3.10. Statistical Analysis

All the results needed more than 3 experiments. The experimental design of the response surface analysis was established by using Design-Expert V13.0.1.5 software to improve the extraction method. SPSS statistical V27.0.1 software was used for statistical analysis. The difference was statistically significant when *p* < 0.05.

## 4. Conclusions

In summary, the ultrasonic-assisted enzymatic method was used to extract bound polyphenols from *E. angustifolia* L., and the key parameters were optimized by the Box–Behnken design. The optimum conditions were as follows: enzyme ratio (α-amylase/cellulase = 5:1 mg/mg), ultrasonic time of 50 min, liquid-to-solid ratio of 12:1 mL/g, and pH value of 5. Under these conditions, the bound polyphenol yield was measured at 13.970 ± 0.3 mg/g. A total of 27 phenolic compounds were identified by UPLC–IMS-QTOF-MS. Finally, through DPPH and ABTS antioxidant tests, when the additional amount of BP extract was 200 μL/mL, the scavenging abilities of DPPH and ABTS were as high as 989.28 and 957.86 μmol Trolox/L. This indicates that *E. angustifolia* L. BP has good antioxidant activity. This study increased our understanding of the medicinal and edible value of *E. angustifolia* L., provided an improved theoretical basis for the most effective use of *E. angustifolia* L., and provided potential functional food ingredients for the food industry. However, further research is required to delve deeper into the antioxidant and other biological activities of *E. angustifolia* L. BPs, as well as their specific mechanisms of action and biological accessibility within the human body.

## Figures and Tables

**Table 1:** Response surface test design and results.

No.	A	B	C	D	Y (mg/g)
1	1	1	0	0	12.598
2	0	1	0	1	12.850
3	0	−1	−1	0	12.499
4	0	1	0	−1	12.268
5	0	0	0	0	14.270
6	−1	0	−1	0	13.000
7	−1	0	1	0	12.224
8	1	0	1	0	10.544
9	1	0	0	−1	12.100
10	−1	0	0	1	12.744
11	1	0	0	1	11.137
12	−1	1	0	0	12.645
13	0	−1	0	−1	12.356
14	0	−1	1	0	11.287
15	0	0	0	0	13.677
16	0	0	−1	−1	12.477
17	0	0	0	0	13.970
18	1	0	−1	0	12.532
19	0	0	1	1	11.551
20	0	1	−1	0	12.470
21	−1	−1	0	0	12.063
22	−1	0	0	−1	11.313
23	0	0	0	0	13.853
24	0	−1	0	1	12.082
25	0	0	0	0	14.138
26	1	−1	0	0	11.657
27	0	1	1	0	12.492
28	0	0	1	−1	12.389
29	0	0	−1	1	12.627

**Table 2:** Analysis of variance for regression variance.

Source	Sum of Squares	Degree of Freedom	Mean Square	F-Value	*p*-Value	Significance
Model	21.36	14	1.53	12.68	<0.0001	**
A	0.9759	1	0.9759	8.11	0.0129	*
B	0.951	1	0.951	7.9	0.0139	*
C	2.18	1	2.18	18.13	0.0008	**
D	0.0006	1	0.0006	0.0053	0.9428	
AB	0.0322	1	0.0322	0.2673	0.6132	
AC	0.3669	1	0.3669	3.05	0.1026	
AD	1.43	1	1.43	11.91	0.0039	**
BC	0.3803	1	0.3803	3.16	0.0971	
BC	0.1834	1	0.1834	1.52	0.2373	
CD	0.2441	1	0.2441	2.03	0.1762	
A^2^	7.83	1	7.83	65.11	<0.0001	**
B^2^	3.75	1	3.75	31.17	<0.0001	**
C^2^	5.34	1	5.34	44.37	<0.0001	**
D^2^	5.63	1	5.63	46.82	<0.0001	**
Residual	1.68	14	0.1203			
Misfit term	1.4	10	0.1402	1.99	0.2654	
Pure error	0.2822	4	0.0706			
Summation	23.04	28				

Note: ** is very significant (*p* < 0.01), * is significant (*p* < 0.05).

**Table 3:** Standard regression equation and correlation coefficient of phenols.

	Regression Equation	Correlation Coefficient
Total phenols	y = 1.8223x + 0.0579	0.9955
Flavone	y = 1.1897x + 0.0007	0.9990
Proanthocyanidins	y = 0.572x − 0.0006	0.9961
Phenolic acid	y = 1.0768x + 0.0901	0.9976
Tannin	y = 1.458x + 0.024	0.9984

**Table 4:** Identification and analysis results of chemical constituents of *E. angustifolia* L. extract.

ID	RT (min)	Chemical Formula	First-Order Mass Spectrometry		Secondary Fragments (*m*/*z*)	Component Name	Type
			Neutral Mass (*m*/*z*)	Observed (*m*/*z*)			
1	2.66	C_9_H_8_O_2_	148.0523	147.045	103.0546	3,4-Dihydrocoumarin	Coumarin
2	2.76	C_16_H_14_O_3_	226.0952	225.0879	147.0448	4′-Methylpinosylvin	Lignin
3	3.46		482.1894	527.1876	271.1294 331.1504 395.1465	2-Butenoic acid, 2-methyl-, (6R,7R,8R,14aS)-5,6,7,8-tetrahydro-1,2-dimethoxy-6,7-dimethyl-3-oxo-3H,14H-benzo [1,8] cycloocta [1,2,3-cd] [1,3] dioxolo [4,5-g] benzofuran-8-yl ester, (2Z)-rel-(-)	Lignin
4	3.62	C_17_H_16_O_2_	252.1106	251.1033	221.0924	Effusol	Polyphenol
5	4.06	C_19_H_18_O_3_	294.1216	293.1143	275.1032	1,7-Bis (4-hydroxyphenyl) hepta-4,6-dien-3-one	Flavone
6	4.28	C_25_H_30_O_8_	358.1732	403.1714	241.1191	Kadsurenin C	Lignin
7	4.98	C_29_H_38_O_9_	530.2535	531.2607	349.0756	Angeloylgomisin Q	Lignin
8	4.67	C_20_H_24_O_3_	312.1771	335.1663	119.0494	Yakuchinone A	Polyphenol
9	6.18	C_9_H_10_O_3_	166.0626	165.0553	120.0214 121.0289	Paeonol	Polyphenol
10	6.23		374.1686	373.1613	357.0982	Cubeb oleoresin	Lignin
11	7.23	C_30_H_34_O_8_	522.2272	523.2345	221.1184	Benzoylgomisin H	Polyphenol
12	9.09	C_18_H_18_O_2_	266.1297	284.1635	221.0928	Juncusol	Polyphenol
13	11.22		446.1216	445.1143	121.0292 313.0719 401.0882	3′-Methoxydaidzein	Flavone
14	12.11	C_19_H_14_O_7_	356.09	401.0882	121.0293 313.0720	3-(1,3-Benzodioxol-5-ylmethyl)-3,4-dihydro-5,7-dihydroxy-8-methyl-4-oxo-2H-1-benzopyran-6-carboxaldehyde	Flavone
15	13.15	C_16_H_18_O_5_	290.113	289.1057	207.1029	5-O-Methylvisamminol	Flavone
16	13.32	C_10_H_10_O_4_	194.0615	239.0597	116.9948	Kakoul	Coumarin
17	13.37		342.0738	341.0665	109.0289 177.0556	3,8-Dihydroxy-4,10-dimethoxy-7-oxo-[2] benzopyrano [2] [1]benzopyran-7-(5H)-one	Polyphenol
18	13.71	C_14_H_14_O_6_	250.0841	249.0767	132.9904 165.0165 177.0921	3-Acetyl-3,4-dihydro-5,6-dimethoxy-2 (1) H-benzopyranone	Coumarin
19	13.95	C_32_H_28_O_11_	600.1668	599.1595	221.1184	Neosappanone A	Polyphenol
20	15.54	C_15_H_14_O_6_	594.1373	593.13	549.1037	Epiafzelechin-(2β-O-7,4β-8)-ent-Epicatechin	Flavone
21	15.6	C_30_H_26_O_13_	594.1403	595.1476	112.9853	Tiliroside	Flavone
22	15.69	C_18_H_16_O_7_	328.0951	327.0878	121.0291	5-Hydroxy-3′,4′,7-trimethoxyflavone	Flavone
23	16.38	C_27_H_22_O_19_	650.0752	649.0679	426.9899	Furosin	Tannin
24	17.47	C_20_H_20_O_5_	340.1305	339.1232	277.0829 291.0989	6,7-dimethoxy-2-(2-(4-methoxyphenyl)ethyl)chromone	Flavone
25	17.8	C_17_H_22_O_4_	292.1678	291.1605	245.1545	6-Dehydrogingerdione	Polyphenol
26	20.6	C_14_H_22_O	206.1671	205.1599	189.1281	2-Octylphenol	Polyphenol
27	26.08	C_21_H_26_O_10_	438.1507	437.1434	183.0118 195.0626	Sec-O-Glucosylhamaudol	Polyphenol

**Table 5:** Factors and levels of response surface design.

Factor		Levels		
		−1	0	1
Enzyme ratio (mg/mg)	A	3:1	5:1	7:1
Time(min)	B	30	50	70
Liquid-to-solid ratio(mL/g)	C	8	12	16
pH	D	4	5	6

## Data Availability

The original contributions presented in the study are included in the article, further inquiries can be directed to the corresponding author.
